# A Microsoft-Excel-based tool for running and critically appraising network meta-analyses—an overview and application of NetMetaXL

**DOI:** 10.1186/2046-4053-3-110

**Published:** 2014-09-29

**Authors:** Stephen Brown, Brian Hutton, Tammy Clifford, Doug Coyle, Daniel Grima, George Wells, Chris Cameron

**Affiliations:** 1 Cornerstone Research Group, Suite 204, 3228 South Service Road, Burlington, ON L7N 3H8, Canada; 2 Ottawa Hospital Research Institute, Center for Practice Changing Research Building, Ottawa Hospital—General Campus, PO Box 201B, Ottawa, ON K1H 8L6, Canada; 3 Canadian Agency for Drugs and Technologies in Health, 865 Carling Ave., Suite 600, Ottawa, ON K1S 5S8, Canada; 4 Department of Epidemiology and Community Medicine, University of Ottawa, 791 of Ottawa, 451 Smyth Road, Suite RGN 3105-H, Ottawa, ON K1H 8M5, 792, Canada; 5 Ottawa Heart Institute, Department of Epidemiology and Community Medicine, University of Ottawa, 40 Ruskin Street, Ottawa, ON K1Y 4W7, Canada

**Keywords:** Network meta-analysis, Software, Microsoft Excel, WinBUGS, Systematic review, Health technology assessment

## Abstract

**Background:**

The use of network meta-analysis has increased dramatically in recent years. WinBUGS, a freely available Bayesian software package, has been the most widely used software package to conduct network meta-analyses. However, the learning curve for WinBUGS can be daunting, especially for new users. Furthermore, critical appraisal of network meta-analyses conducted in WinBUGS can be challenging given its limited data manipulation capabilities and the fact that generation of graphical output from network meta-analyses often relies on different software packages than the analyses themselves.

**Methods:**

We developed a freely available Microsoft-Excel-based tool called NetMetaXL, programmed in Visual Basic for Applications, which provides an interface for conducting a Bayesian network meta-analysis using WinBUGS from within Microsoft Excel. . This tool allows the user to easily prepare and enter data, set model assumptions, and run the network meta-analysis, with results being automatically displayed in an Excel spreadsheet. It also contains macros that use NetMetaXL’s interface to generate evidence network diagrams, forest plots, league tables of pairwise comparisons, probability plots (rankograms), and inconsistency plots within Microsoft Excel. All figures generated are publication quality, thereby increasing the efficiency of knowledge transfer and manuscript preparation.

**Results:**

We demonstrate the application of NetMetaXL using data from a network meta-analysis published previously which compares combined resynchronization and implantable defibrillator therapy in left ventricular dysfunction. We replicate results from the previous publication while demonstrating result summaries generated by the software.

**Conclusions:**

Use of the freely available NetMetaXL successfully demonstrated its ability to make running network meta-analyses more accessible to novice WinBUGS users by allowing analyses to be conducted entirely within Microsoft Excel. NetMetaXL also allows for more efficient and transparent critical appraisal of network meta-analyses, enhanced standardization of reporting, and integration with health economic evaluations which are frequently Excel-based.

## Background

Meta-analysis is a statistical method commonly used to combine summary estimates of treatment effects from a collection of studies to establish the benefits and harms of competing interventions. An important limitation of standard meta-analysis (i.e., pairwise meta-analysis) is that it compares only two treatments at a time [1]. However, many medical conditions exist for which there are a multitude of possible treatment alternatives. Accordingly, new meta-analytic methods have emerged which permit simultaneous comparison of multiple treatments. This new method is referred to as *network meta-analysis* (NMA) (other terms such as *mixed-treatment comparison meta-analysis* and *multiple treatments meta-analysis* have also been used) [1,2]. Not surprisingly, the increasing need to compare multiple treatments for medical conditions has been mirrored by the dramatic rise in the use of network meta-analysis in recent years [3,4].

Both Frequentist and Bayesian approaches for conducting network meta-analysis are feasible [1,5-7]. Among them, the latter has been the more commonly used framework, likely because the methods have evolved more quickly, because Bayesian methods provide greater flexibility to use more complex models and different outcome types, and because Bayesian methods are easily integrated into health economic evaluations. WinBUGS is a freely available software package available for Bayesian data analysis and has been the most widely used package to conduct network meta-analyses to date [3]. However, the learning curve for using WinBUGS to conduct network meta-analyses successfully can be daunting for new users, given the challenges of understanding WinBUGS code. WinBUGS also has limited data manipulation, data annotation, and graphical illustration capabilities. Given these challenges, the preparation of tables and figures to present insightful summaries of findings from network meta-analyses often requires use of an additional software package [7], thereby adding time and an additional layer of complexity to complete reports. As a result, current approaches do not allow analysts to simply update and reanalyze models quickly and efficiently. Further, the variability in software used and the lack of standardization has led to inconsistency in the reporting of analyses to health technology assessment (HTA) organizations and journals, thereby complicating the review process. Given these challenges, more user-friendly software is needed to facilitate the ability to perform, consistently report, and critically appraise network meta-analyses for a broader group of researchers. More integrated and user-friendly software will dramatically improve the transparency and reproducibility of findings from network meta-analyses.

The objectives of this paper are to use an illustrative example to demonstrate how our Microsoft-Excel-based Network Meta-analysis Tool (NetMetaXL) can be used to simplify running and reporting network meta-analyses and to highlight how NetMetaXL can be used to facilitate consistent reporting and more efficient and transparent critical appraisal of network meta-analyses submitted to HTA organizations such as the Canadian Agency for Drugs and Technologies in Health (CADTH) and the National Institute for Health and Care Excellence (NICE), as well as to journals which publish network meta-analyses.

## Methods

### The illustrative example

Table 1 presents the illustrative dataset derived from a network meta-analysis evaluating combined resynchronization and implantable defibrillator therapy in left ventricular dysfunction [8]. The methods used to identify studies included in the network meta-analysis and to collect trial-level data have been described previously [8]. A total of 12 randomized studies comparing 5 different treatments (medical resynchronization, cardiac resynchronization, implantable defibrillator, combined resynchronization and defibrillator, and amiodarone) were included in the review, encompassing a total of 1,616 deaths in 8,307 participants. The authors conducted a Bayesian random effects network meta-analysis to compare the overall risk of mortality among the different treatments.

**Table 1:** Dataset used for illustrative example

**Study number**	**Study name**	**Medical**		**Cardiac resynchronization**		**Implantable defibrillator**		**Combined resynchronization and defibrillator**		**Amiodarone**	
		**Number of events**	**Number of patients**	**Number of events**	**Number of patients**	**Number of events**	**Number of patients**	**Number of events**	**Number of patients**	**Number of events**	**Number of patients**
1	CARE_HF-ext	154	404	101	409						
2	COMPANION	77	308	131	617			105	595		
3	MIRACLE	16	225	12	228						
4	MUSTIC-SR	0	29	1	29						
5	SCD-HeFT	244	847			182	829			240	845
6	MADIT-II	97	490			105	742				
7	DEFINITE	40	229			28	229				
8	CAT	17	54			13	50				
9	MICRACLE-ICD-I					5	182	4	187		
10	MICRACLE-ICD-II					2	101	2	85		
11	CONTAK-CD					16	245	11	245		
12	AMIOVIRT					6	51			7	52

The data is derived from Lam and Owen [8].

### Application of NetMetaXL using an illustrative example

This tool was designed to allow users to run network meta-analyses, as well as to appraise Bayesian network meta-analyses using WinBUGS via a more user-friendly Microsoft Excel interface. The current versions of NetMetaXL only allow the user to apply Bayesian network meta-analysis for binomial data and logistic regression models. This section describes how users can use this tool in the context of the illustrative example above. It is critical that users of NetMetaXL receive training on network meta-analysis. Users should be educated on key concepts related to network meta-analysis and how to interpret findings for decision-making purposes. Users are also encouraged to consult with a statistician when using this tool.

### Step 1: Software installation

The application of NetMetaXL requires installation of Microsoft Excel 2007 or higher, as well as installation of the WinBUGS 1.4.3 package. NetMetaXL will work using Windows XP, Windows 7, or Windows 8. Before opening NetMetaXL, the user will install the WinBUGS 1.4.3 package and download and install the patch for WinBUGS 1.4.3 and the key for unrestricted use as described on the WinBUGS website. Detailed instructions on installing WinBUGS are available on the WinBUGS website. After the user has installed WinBUGS, the user can download the latest version of the NetMetaXL package from http://www.NetMetaXL.com. Any user can download the tool and administrator access is required. This Excel-based tool is also part of the CADTH online repository of Microsoft-based tools for enhancing the application of HTAs: http://www.cadth.ca/en/resources/hta-excel-tools. NetMetaXL is programmed in Visual Basic for Applications within Excel and links to WinBUGS using Visual Basic. After NetMetaXL and WinBUGS have been downloaded and installed, the user will open NetMetaXL and go to the WinBUGS Setting tab visible in the menu bar at the top of the screen. Within this tab, under Program Settings, the user will indicate where the WinBUGS executable file that was downloaded is located on their computer’s hard drive. For example, the user can click the file folder icon next to the WinBUGS Directory (cell D41) and then browse to specify the location of the WinBUGS Directory (e.g., C:\Program Files\WinBUGS14\).

### Step 2: Setup of specifications for analysis

After NetMetaXL has been installed and is linked to WinBUGS, the user is ready to begin conducting or appraising network meta-analyses. The user should then open the file and save it using a study-specific name. Within NetMetaXL, the user will see a WinBUGS menu bar in the top right of Microsoft Excel. WinBUGS is a software for conducting Bayesian analysis using Markov chain Monte Carlo simulation [9]. A Bayesian analysis using WinBUGS requires two main ingredients: prior distributions for the unknown parameters and a likelihood function derived from a model that specifies the relation between the unknown parameters and the observed data [10,11]. A prior distribution of a parameter represents the uncertainty about the parameter before the current data are examined [10,12-16]. The prior chosen may be informative or ‘vague.’ The latter is thought to ‘let the data drive the analysis,’ but the use of a vague prior should not be used unthinkingly especially when data is sparse because a vague prior may actually influence the analysis [10,12-16]. Multiplying the prior and the likelihood function together leads to the posterior distribution of the parameter. The posterior distribution is used to carry out all inferences [10,12-16].

To run the Bayesian analysis using WinBUGS, a series of procedures are required, all of which are automated within NetMetaXL. In particular, the user must check that the model is properly specified, load the data, and select the number of chains (or samples) to specify the initial values for certain parameter estimates; set up monitors to store the sampled parameter values; run the simulation; check convergence for the parameter estimates; and then obtain a summary of the posterior distribution of the selected parameter estimates.

The user will also see a number of worksheets, each devoted to different aspects of preparing the data for Bayesian analysis and generating graphical summaries of the results after the analysis has been run. The majority of settings for the setup of specifications for analysis are located within the WinBUGS Settings worksheet. Within this worksheet, the user specifies analysis characteristics such as WinBUGS Settings (e.g., number of burn-in iterations for assessing convergence of parameter estimates); statistical settings such as which model parameters to capture (e.g., odds ratio, treatment rankings), whether the outcome being considered is ‘bad’ (e.g., dead at the end of the study) or ‘good’ (e.g., alive at the end of the study) from the patient’s perspective, informative prior settings (NetMetaXL applies vague priors for log odds ratios but allows the user to use informative priors for between-study heterogeneity variances derived from based on a publication by Turner et al. [14] which is potentially useful when data is sparse), and initial values for parameter estimates (NetMetaXL selects these randomly from a uniform distribution within the ranges chosen for log odds ratios and log odds) which should be checked in WinBUGS data sheet; and Program Settings (e.g., location to save files).

### Step 3: Data input

After the user specifies the WinBUGS and statistical settings on the WinBUGS Settings worksheet, the dataset for analysis in NetMetaXL can next be entered. To input the data, the user selects the Data Input tab. A screenshot displaying the dataset is presented in Table 1. An additional file showing the screenshot with the dataset in NetMetaXL is also provided [see Additional file 1]. To begin inserting the data, the user must specify the name of each treatment under consideration in row 5; the tool has currently been designed to handle up to 15 interventions of interest and up to 50 studies. The choice of treatment 1, also known as the reference treatment, is an important consideration. Ideally, the user will select the treatment with the most studies as the reference treatment. After the user specifies the treatment names, the user inserts the number of observed events and total sample size for the intervention groups in each study beginning in row 7. In accordance with the most commonly used Bayesian implementation of network meta-analysis, users are able to input more than two treatment groups for a given study and the software will account for correlation between trial arms. As the user inputs the data for all studies and treatments, cells C2–C5 will update automatically to reflect properties of the evidence base under study in terms of the total number of included studies and patients in the treatment network. In the case of our illustrative example, once data input is complete, cells C2–C5 will indicate that the evidence base consists of 12 studies and 5 treatments and will also indicate that 8 of the included studies include medical therapy as one of their treatment arms (our chosen control treatment, labeled as treatment 1 in our network). After the data is inserted, the user is ready to begin running network meta-analyses using NetMetaXL.

### Step 4: Preparing the data for WinBUGS

There is a ‘Convert Data’ button within the menu bar. When the user selects this button, there will be a prompt asking: ‘Correct for zero values?’. If the user selects, ‘Yes’, NetMetaXL will adjust all zero cells using an adjusted continuity correction factor accounting for potential differences in sample size and centered around 0.5 [16]. If the user selects ‘No’, zero cells will be included in the analysis and no adjustment is applied. An advantage of the Bayesian approach is that special precautions do not usually need to be taken in the case of the occasional trial with a zero cell count [17]. NetMetaXL allows users the run the analysis with zeroes when there are occasional zero cells and attempt to resolve the issue by adjusting otherwise, although in some cases the results will remain unstable even after adjusting [17]. The zero cell correction within NetMetaXL keeps all studies for analytic consistency (i.e., does not delete studies entered by the user) even those with multiple zero cells although these do not contribute to the relative effect estimation and can cause computational problems when networks are sparse. Because studies with zero cells remain, model fit statistics for these analyses should be interpreted with caution given they make certain model fit statistics artificially look better. The users of this tool should always consult with a statistician, but especially when dealing with zero cells. After the user selects either of these choices, NetMetaXL will convert the data provided into the appropriate format for analysis within WinBUGS. WinBUGS requires the data to be in a specific format and for the user to specify the initial values. The data after the conversion is reported within the WinBUGS data tab. NetMetaXL will also perform this step automatically when pushing the ‘Run WinBUGS’ button.

## Results

There are key outputs to consider when interpreting a network meta-analysis. Notably, the user should carefully review the geometry of the evidence network, which provides information related to the number of studies performed comparing the different treatments, the numbers of patients who have been studied for each treatment, and so forth. After ensuring convergence has been reached and there is no relevant inconsistency in the evidence, one can use the output from the consistency model to draw conclusions about the relative effects of treatments [18]. This information is often displayed in tabular or graphical format such as a forest plot or league table. Alternatively, information of relative effects is sometimes converted to a probability a treatment is best, second best, and so on, or the ranking of each treatment. More recently, these two measures have been combined into a single measure called the surface under the cumulative ranking curve (SUCRA) [18], which is expressed as a percentage—the SUCRA would be 100% when a treatment is certain to be the best and 0% when a treatment is certain to be the worst. The outputs for these elements in NetMetaXL are described in steps 5–9 below.

### Step 5: Visualizing the treatment network

**Figure 1:**
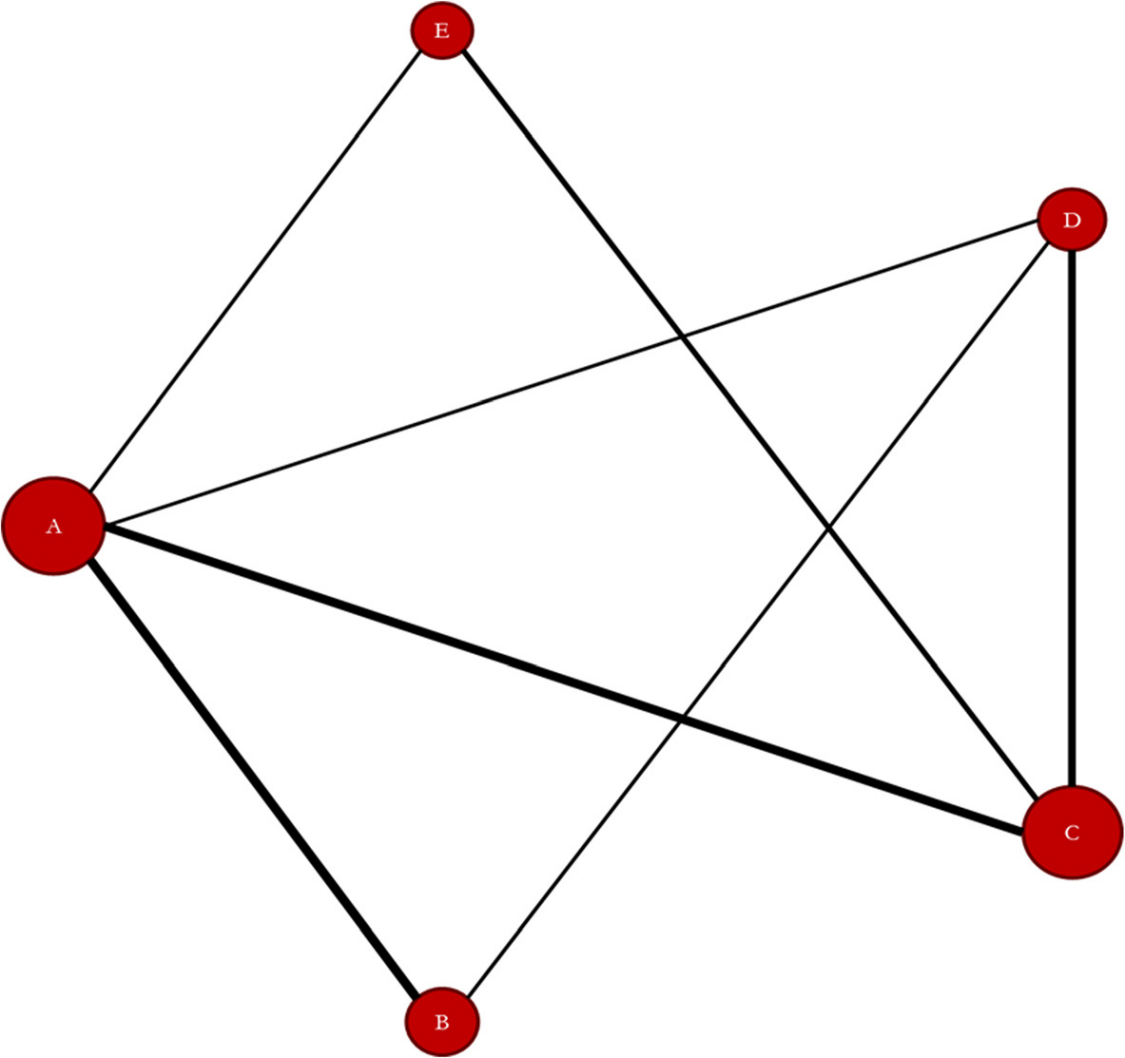
**Evidence network diagram.***A* medical therapy, *B* cardiac resynchronization, *C* implantable defibrillator, *D* combined resynchronization and defibrillator, *E* amiodarone.

After the user selects the WinBUGS tab from the menu bar in NetMetaXL, the user can generate a treatment network diagram by selecting the Generate Diagram button. After the user clicks this button, an evidence network will be generated (see Figure 1). As has become common practice in reports of network meta-analyses [7], the width of each edge in the evidence network is proportional to the number of randomized controlled trials comparing each pair of treatments, and the size of each treatment node is proportional to the number of randomized participants (sample size). A tabular description of the evidence network is also provided within the Data Summary worksheet. The user has the capability to indicate the treatment names to be used on the network diagram in cells C25–C29. For example, instead of labeling the nodes simply as A–E as in Additional files 1 and 2, the user can input the treatment names for each node (or preferred abbreviations which are a preferable option when dealing with longer treatment names). After the labels are changed in cells C25–C29, the user can push the Generate Diagram button again from the WinBUGS tab in the toolbar, and the names in cells C25–C29 will appear within the network diagram. The nodes can also be moved, if desired, and the connections can automatically be redrawn by clicking the ReDraw Connections button. The user can copy the evidence network by holding control and selecting the nodes and connections and subsequently pasting the evidence network into other programs such as PowerPoint.

### Step 6: Running network meta-analyses using NetMetaXL

**Figure 2:**
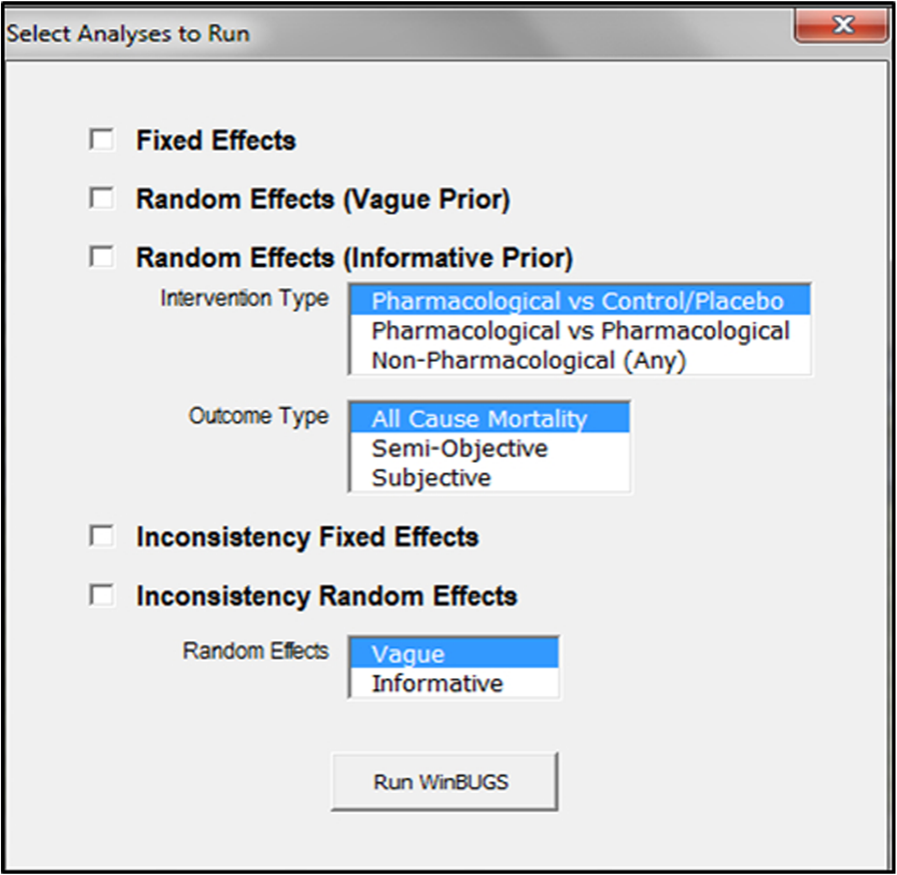
Analysis dialog box from NetMetaXL.

To run network meta-analyses within NetMetaXL, the user selects the Run WinBUGS button within the menu bar. After selecting the Run WinBUGS button, NetMetaXL will open WinBUGS to run the network meta-analyses and will then close WinBUGS after transferring back to NetMetaXL the results for all the prespecified parameters of interest specified in the WinBUGS Settings tab during the setup of the analysis in WinBUGS Settings tab. These analysis functions and others are provided within the WinBUGS tab within the menu bar in the upper right of NetMetaXL. NetMetaXL generates commonly used graphical summaries of results including forest plots, league tables, and probability bar plots (or rankograms); a separate worksheet is devoted to each type of graphical output. To begin the analyses, the user clicks the Run WinBUGS button from the WinBUGS tab in the menu bar of NetMetaXL once data have been appropriately formatted as outlined in step 3. After the user clicks this button, a dialog box will open where the user will select the different network meta-analyses to be conducted (Figure 2). For example, the user can run analyses using a fixed effects model, a random effects model using vague priors as outlined in NICE Evidence Synthesis Series [17], and/or a random effects model using informative variance priors [14]. For the random effects model using vague priors, we assume use the following prior: sd ~ dunif(0,2). For the random effects model using informative variance priors, the user indicates the type of outcome under consideration as well as the type of treatment (e.g., placebo, active pharmacological treatment). NetMetaXL uses these selections and bases the informative variance priors on evidence on the extent of heterogeneity observed in previous meta-analyses, as described in Turner et al. [14] For all analyses, we assume vague priors on baselines [dnorm(0,10000)] and basic parameters [dnorm(0,10000)]. After the user selects the analyses to be conducted using the check boxes within the dialog box, the user then selects Run WinBUGS from within the dialog box; this will launch WinBUGS and will automatically run analyses and import results based on the various models selected back into NetMetaXL in fixed effects (FE) Results or random effects (RE) Results worksheets. The WinBUGS results are also saved as an *.odc file in the directory specified in the Program Settings (WinBUGS Setting tab). The WinBUGS code used for generating all the network meta-analyses is based on the NICE Decision Support Unit Series [5,17]. The WinBUGS model codes are stored within NetMetaXL on the worksheets titled FE model, RE Model, RE Inconsistency Model, and FE Inconsistency Model. The user also has the option to open WinBUGS directly and examine the underlying WinBUGS code if need be using the Open WinBUGS button in the toolbar.

NetMetaXL captures all the WinBUGS output [see Additional file 2] and stores them in FE Results or RE Results worksheets. NetMetaXL will capture output results from WinBUGS for any parameters selected in the WinBUGS Settings tab (e.g., odds ratios). The user should refer to FE and RE model tabs to see the statistics behind the parameter calculations.

### Step 7: Checking convergence in NetMetaXL

We fit three chains for the Markov chain Monte Carlo (MCMC) Bayesian network meta-analysis. The use of multiple chains is a useful way to check MCMC convergence. The user selects the initial values for each of these chains randomly from a uniform distribution. The user can select the bounds for the uniform distribution in the WinBUGS Settings worksheet. The user is encouraged to review the following paper [19] for additional detail on selecting initial values. Convergence is assessed in NetMetaXL using the Brooks-Gelman-Rubin method and by checking whether the Monte Carlo error is less than 5% of the sd of the effect estimates and between-study variance. These diagnostics are provided when the user runs the analysis. NetMetaXL will check whether the Monte Carlo error is less than 5% of the sd of the effect estimates and between-study variance and gives the user the option to view the Brooks-Gelman-Rubin plots from the WinBUGS output. The Brooks-Gelman-Rubin method compares within-chain and between-chain variances to calculate the potential scale reduction factor [20]. A potential scale reduction factor is presented in red in the figure, and a value close to one indicates when approximate convergence has been reached.

### Step 8: Generating a graphical summary of findings from network meta-analysis

**Figure 3:**
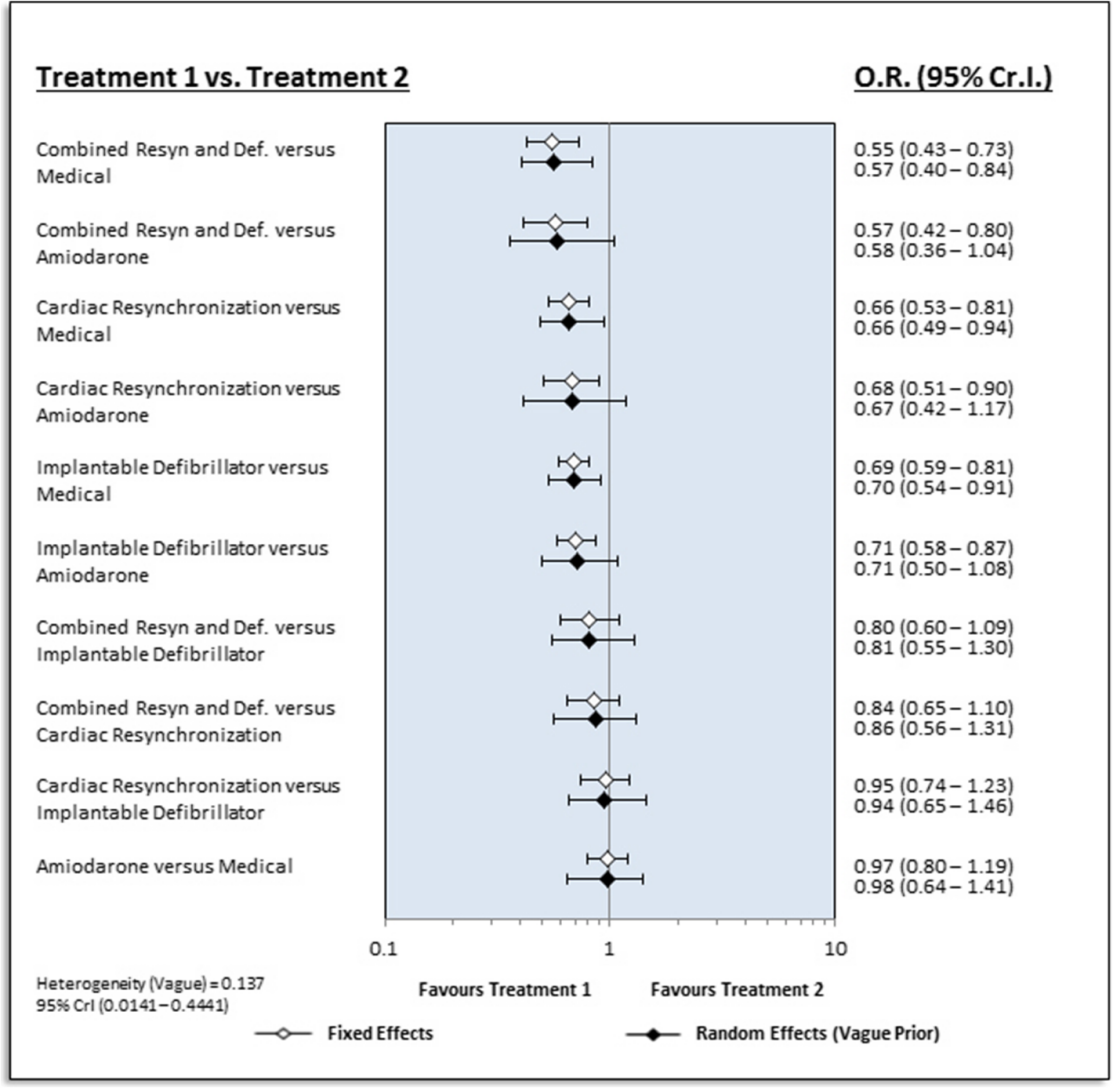
Forest plot from NetMetaXL.

NetMetaXL captures all the WinBUGS output and then uses VBA macros to construct different graphical representations common to reports of network meta-analyses [18]. NetMetaXL is capable of generating forest plots and league tables to summarize all pairwise comparisons between the competing treatments, as well as probability bar plots (or rankograms). A forest plot generated by NetMetaXL for our illustrative example is presented in Figure 3. On the forest plot worksheet, the user can select the analyses they would like reported in the forest plot. For example, the user can present findings for only one model (e.g., fixed effects model) or can also choose to report findings using both the fixed and random effects models. The user can also select the spacing, marker size, and plot size in cells D7–D9 to maximize the quality of the figure. The forest plots then illustrate the median effect estimate for each pairwise comparison within the network meta-analysis for each model fit, along with corresponding 95% credible intervals.

**Figure 4:**
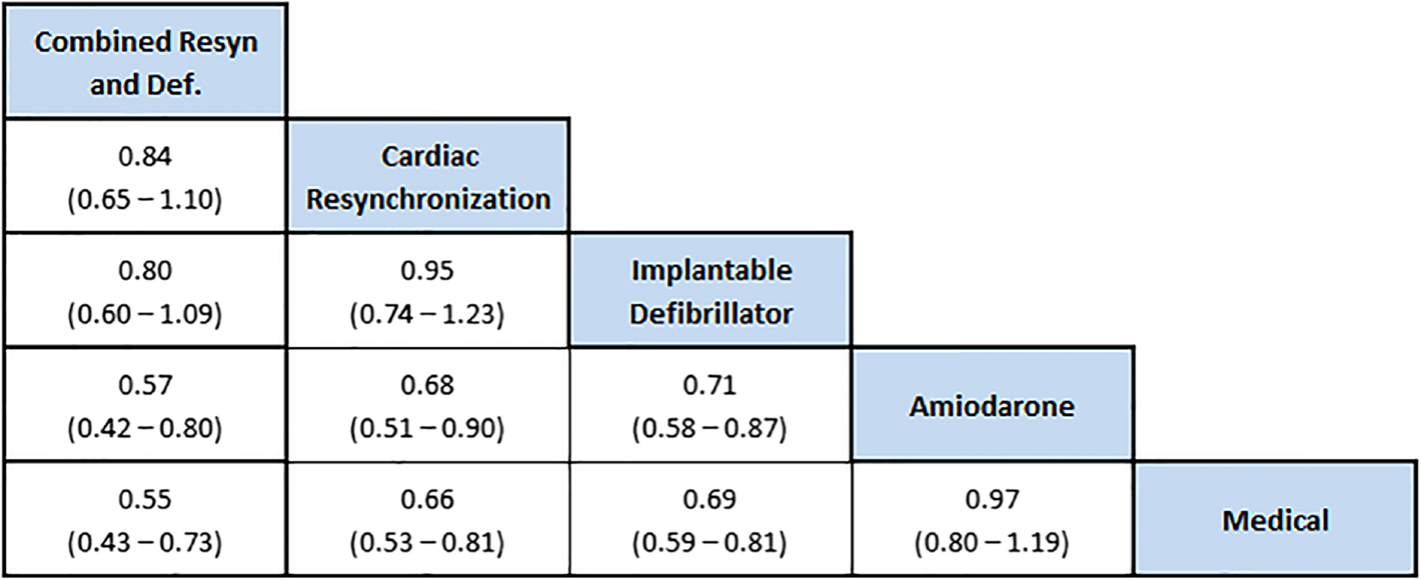
League table from NetMetaXL.

In addition to generating forest plots, the user can also generate league tables within NetMetaXL to summarize all possible pairwise comparisons between the interventions. The summary league table for our illustrative example is shown in Figure 4. The league table arranges the presentation of summary estimates by ranking the treatments in order of most pronounced impact on the outcome under consideration, based on SUCRA [18]. SUCRA, the surface under the cumulative ranking [18], is a simple numerical summary of the probabilities. It is 100% when a treatment is certain to be the best and 0% when a treatment is certain to be the worst. SUCRA values enable the ranking of treatments overall for a particular outcome. For example, in our illustrative example, combined resynchronization and defibrillation is listed in the top left of the diagonal of the league table because it was associated with the most favorable SUCRA for mortality reduction, while medical therapy is listed in the bottom right of the diagonal of the league table because it was associated with the least favorable results. For interpretation purposes, the results are read from top to bottom and left to right. For example, using the random effects model with vague priors (as was used in Lam and Owen [8]), combined resynchronization and defibrillation compared with cardiac synchronization is associated with an odds ratio of 0.84 (0.57–1.22), suggesting it is trending towards being better than cardiac synchronization in terms of mortality, whereas cardiac resynchronization versus medical therapy is associated with an odds ratio of 0.66 (0.50–0.89).

Probability bars (or rankograms) [18] are also generated and reported within NetMetaXL, which report the probability that each treatment is ranked first, second, and so on for a particular outcome. These rankograms are depicted as stacked vertical bar charts within NetMetaXL for all treatments. The user can also output line graphs for all treatments or generate bar charts or line graphs for individual treatments.

### Step 9: Assessment of inconsistency

**Figure 5:**
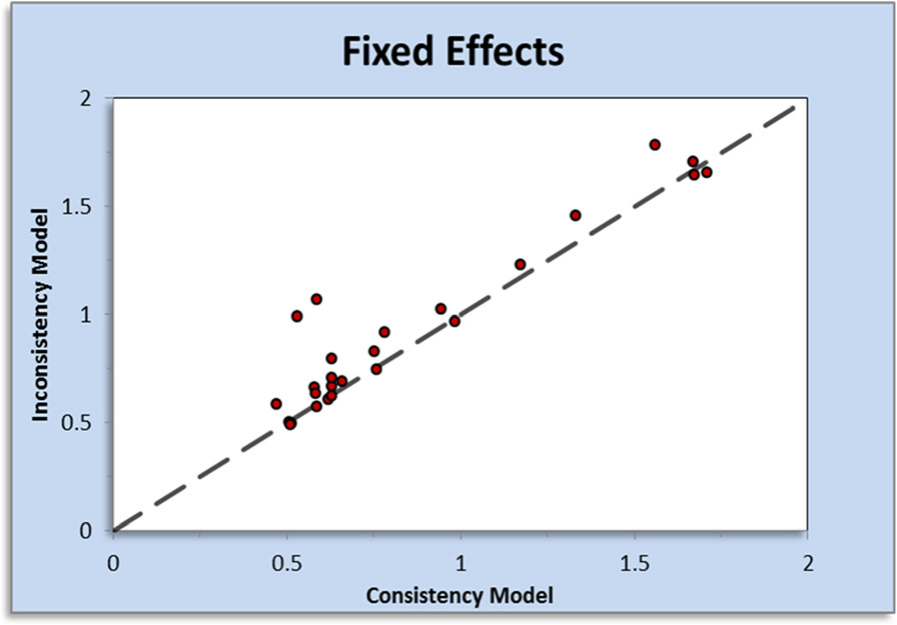
Inconsistency plot from NetMetaXL.

Assessment of inconsistency is crucial in the conduct of any network meta-analysis. Inconsistency can be thought of as a conflict between ‘direct’ and ‘indirect’ evidence [5]. Similar to heterogeneity, inconsistency is caused by imbalances in effect modifiers from study to study, specifically by an imbalance in the distribution of effect modifiers in the direct and indirect evidence [5]. NetMetaXL allows users to assess inconsistency by comparing the deviance residuals and DIC statistics in fitted consistency and inconsistency models [5]. These are reported in a table in the Inconsistency results worksheet. We refer the readers to the NICE Technical Support Documents (TSD) series for the methods employed [5]. NetMetaXL also plots the posterior mean deviance of the individual data points in the inconsistency model against their posterior mean deviance in the consistency model to identify any loops in the treatment network where inconsistency is present (Figure 5) [5]. NetMetaXL also allows the user to select the points on the inconsistency plot and see which study and treatment is represented by that point. For example, by double-clicking on the point in Figure 5 within NetMetaXL, we see that this point is from the cardiac resynchronization arm of the COMPANION study. This feature will be particularly useful for network meta-analyses where there are a number of points in the bottom right of the inconsistency plot, indicating potential inconsistency [5].

## Discussion

### Summary of main findings

We have shown how use of NetMetaXL can enhance the ability of users to run WinBUGS-based network meta-analyses entirely within Microsoft Excel. We have replicated findings from a network meta-analysis [8] published in the BMJ on combined resynchronization and implantable defibrillator therapy in left ventricular dysfunction using NetMetaXL. The approach and steps used in this illustrative example can be applied to running other network meta-analyses of dichotomous outcomes entirely through the Microsoft-Excel-based NetMetaXL tool, without requiring the user to directly use the WinBUGS software. In the future, we plan on developing similar tools for other outcome measures.

There are several software packages available to run network meta-analyses. The majority of network meta-analyses conducted to date have used WinBUGS [3], although new routines have been developed which allow network meta-analyses to be conducted with STATA [6,7,21], R [22], and SAS [23]. These approaches to conducting network meta-analyses share a similar feature—they require programming knowledge of the software package being used. This is not a problem for statisticians who use these packages regularly or reviewers who are well versed in network meta-analyses and these software. However, this is a challenge for non-statisticians or reviewers who are not familiar with the packages but would like to run or critically appraise a network meta-analysis. Some of these packages (e.g., WinBUGS) have poor data manipulation functionality, data annotation, and graphical illustration capabilities, thereby requiring results to be transferred between multiple software packages, adding an additional layer of complexity.

This has led to the development of more user-friendly and integrated packages such as the Aggregate Data Drug Information System (ADDIS) [24]. Similar to our NetMetaXL tool, ADDIS [24] also provides users with a more user-friendly software package to run network meta-analyses without directly using WinBUGS (or JAGS or OpenBUGS) code. However, the current version of ADDIS (ADDIS 1) uses a stand-alone software package. Using Microsoft Excel offers some advantages over a stand-alone package like ADDIS such as the following: 1) it allows users to use a software that they are familiar with, 2) there is more potential for others to develop add-ons to the Excel-based tool (versus stand-alone package) given the wide user base, and 3) it allows the data and results to be more easily integrated with decision analytic models and health economic evaluations which are frequently Excel-based compared to stand-alone packages. Indeed, this was noted in a recent publication [25] where they developed an Excel-based tool to perform HTAs entirely within Microsoft Excel. This Excel-based tool developed by Bujkiewicz et al. [25] called the transparent interactive decision interrogator (TIDI) also integrated with WinBUGS but used R, another software with a steep learning curve for non-statisticians. Although the example in TIDI was not specific to network meta-analysis, TIDI [25] could also be used for conducting and critically appraising network meta-analyses as well. Despite the advantages of using Excel, there are also disadvantages. Excel is a free-form tool and accordingly there are opportunities for both user and programmer error. Users should double- and triple-check data inserted into NetMetaXL. To reduce the risk of programmer error, we used standard WinBUGS code provided in the NICE TSD series and had an independent statistician review the WinBUGS coding.

### Application of NetMetaXL

Network meta-analysis is increasingly being used to provide estimates of effect for treatments that may not have been compared directly in clinical trials. NetMetaXL represents a step forward in improving the ability of novice users to run network meta-analyses. We have illustrated this application for running network meta-analyses in NetMetaXL using the combined resynchronization and implantable defibrillator therapy illustrative example. Another useful application of NetMetaXL would be related to critical appraisal of network meta-analyses by HTA organizations such as CADTH or NICE, or network meta-analyses submitted to journals. Inclusion of a NetMetaXL file, along with a technical report or publication, would undoubtedly facilitate more efficient and transparent critical appraisal of network meta-analyses. In addition, it would provide some standardization to the format of the analysis and graphical reporting.

HTA organizations and major medical journals should encourage authors to submit the data for their systematic reviews and network meta-analyses using NetMetaXL or a similar software package. Currently, the data used in HTAs or published network meta-analyses is often not adequately reported, presented in a wide range of styles, or often embedded in images such as forest plots, making it challenging to extract data quickly, replicate findings, and critically appraise the study. The use of data repositories such as the Systematic Review Data Repository [26] and the Dryad Digital Repository has improved accessibility of data. However, these repositories only require that the data and code are uploaded to the repository and are often made available for use in less user-friendly packages such as R, STATA, or SAS. These repositories do not require that data are formally integrated with the analysis. Accordingly, critical appraisal of network meta-analyses, especially for non-statisticians, is still a challenge even when data is submitted to these repositories because users are still often left without the tools to quickly validate the analyses. By contrast, NetMetaXL will allow users to quickly validate the submitted analysis and test robustness of results to excluding certain studies, use of informative/non-informative priors, and choice of fixed/random effects models.

Some organizations in the healthcare sector have recognized the limitation of not providing the unified data and model on which an analysis is based. Indeed, HTA organizations often require manufacturers of drugs and devices to provide a user-friendly health economic model, in specified software, in addition to a technical report to facilitate critical appraisal of their product; however, this is not the standard for network meta-analyses submitted to HTA organizations. These health economic models are often Microsoft Excel based and give reviewers much more information than is provided in a 100-page technical report, let alone a 2,700–4,000-word publication. Given that type of easy access to and control of the original authors’ data, model, and analysis, reviewers at HTA organizations are able to run additional analyses to test model uncertainty and alternative assumptions. The impact that these Microsoft-Excel-based models have had on critical appraisal of manufacturers’ health economic models is evident by examining the public summary documents from HTA organizations such as CADTH and NICE. It is not uncommon for recommendations from HTA bodies to report that reanalyses conducted by their staff found the cost-effectiveness estimates were less favorable than those submitted by the manufacturer. The same impact could potentially be seen if this increased level of transparency was applied to network meta-analyses.

### Limitations of NetMetaXL

There are a number of limitations with NetMetaXL to note. Currently, this version of NetMetaXL only allows users to consider a maximum of 15 treatment options and 50 studies. This will prevent its application to more complex networks with multiple dosing strategies, complex interventions, or well-established disease areas where there are more than 15 treatment options. The current version of NetMetaXL is also only applicable for dichotomous outcomes. However, we will develop similar tools for other outcome types (e.g., continuous), will continue to update and refine the Microsoft-Excel-based tool, and will make new versions freely available online, including additional capabilities to adjust for heterogeneity including meta-regression analysis and inclusion of more sophisticated methods for assessing inconsistency. All versions of NetMetaXL will be housed on http://www.NetMetaXL.com. This tool is the first in a series of Excel-based tools being developed with support from CADTH. These Excel-based tools are being developed to enhance the application of HTAs in Canada and abroad. There will also be a link to NetMetaXL on the PRISMA website to enhance uptake and improve transparency of network meta-analyses in published medical journals.

With availability of user-friendly software such as NetMetaXL, concerns arise about network meta-analysis being undertaken and implemented inappropriately by novice users. It is critical that users of NetMetaXL receive training on network meta-analysis. Users should be educated on key concepts related to network meta-analysis such as heterogeneity and inconsistency and how to interpret findings for decision-making purposes. Users are also encouraged to consult with a statistician when using this tool.

Another disadvantage of NetMetaXL is that Microsoft Excel keeps the data locally (unless integrated with the cloud-based Microsoft 365 or other cloud systems) and does not foster sharing of information/data. This may be advantageous to drug or device manufacturers when submitting confidential data and network meta-analyses to HTA organizations such as CADTH and NICE. However, such an environment is not good for the society as a whole. Rather than keeping data in silos, there is a need to foster a research environment where data are shared and can be dynamically updated by multiple collaborators to improve research productivity. The earlier-mentioned ADDIS software [24] is in the process of launching a web-based platform (ADDIS 2) where researchers can collaborate to perform systematic reviews, data extraction, evidence synthesis, and decision analysis. Undoubtedly, such an integrated system should improve the efficiency, transparency, and critical appraisal of systematic reviews and network meta-analyses dramatically by making data more accessible to researchers and collaboration more seamless. As a result, we are strong ambassadors of the research that ADDIS [24] is striving towards. However, until use of such a system becomes widespread and readily available, NetMetaXL will serve as a step in the right direction towards making the conduct of network meta-analyses more accessible to non-statisticians and facilitating more efficient and transparent critical appraisal of network meta-analyses, standardization in reporting, and more seamless integration with health economic evaluation.

## Conclusions

We have demonstrated the application of a freely available Microsoft-Excel-based tool—NetMetaXL—using an example of a published network meta-analysis. NetMetaXL can make running and reporting network meta-analyses more accessible to novice users, as all aspects of data entry, analysis, and reporting are conducted entirely within Excel and require no specialized programming knowledge. The application of this Microsoft-Excel-based tool could also facilitate more efficient and transparent critical appraisal of network meta-analyses submitted to HTA organizations such as CADTH or NICE, or journals which publish network meta-analyses. The use of this tool may also help standardize reporting and enhance integration with health economic evaluations.

## Competing interests

DG is a partner at Cornerstone Research Group and SB is an employee of Cornerstone Research Group. BH has provided advice to Amgen Canada regarding methodological issues related to network meta-analysis. The authors declare that they have no competing interests.

## Authors’ contributions

CC drafted the manuscript, incorporated comments/feedback received on the manuscript, conceived the study design, validated the statistical/epidemiological analysis, provided the appropriate WinBUGS code to SB, assisted with developing the software package, and submitted the manuscript for publication. SB conceived the study design, wrote all the visual basic codes for running the network meta-analysis, developed the software package, incorporated comments/feedback related to the software, and reviewed the manuscript for important intellectual content. BH piloted NetMetaXL and provided feedback to CC and SB to incorporate into the tool. BH, DC, TC, DG, and GW conceived the study design and reviewed the manuscript for important intellectual content. All authors have read and approved the final version of the manuscript. All authors agree to be accountable for all aspects of the work in ensuring that questions related to the accuracy or integrity of any part of the work are appropriately investigated and resolved.

## Authors’ information

CC and SB are co-first authors. CC drafted the manuscript, validated the statistical/epidemiological analysis, provided the appropriate WinBUGS code to SB, and coordinated the development of the website to download NetMetaXL and track use of the tool; SB wrote all the visual basic codes for running the network meta-analysis, developed NetMetaXL, and reviewed the manuscript for important intellectual content.

## Supplementary Material

Additional file 1 **Screenshots of NetMetaXL.** Multiple screenshots for selected steps within NetMetaXL: data input, generating network diagram, using dialogue box, generating forest plots, generating league tables, and generating inconsistency plots. Click here for file

Additional file 2 **Output from WinBUGS.** Presentation of traditional output from WinBUGS for illustrative example—network meta-analysis evaluating combined resynchronization and implantable defibrillator therapy in left ventricular dysfunction [8]. Click here for file
