# Supplementary material for: A Microsoft-Excel-based tool for running and critically appraising network meta-analyses—an overview and application of NetMetaXL
Source: Syst Rev. 2014 Sep 29;3:110. doi: 10.1186/2046-4053-3-110 (PMC4195340; doi:10.1186/2046-4053-3-110)

**Additional file 1 – Screenshots from NetMetaXL**

**Figure 1:** Screenshot of dataset used for illustrative example. The data is derived from Lam et al.[8]


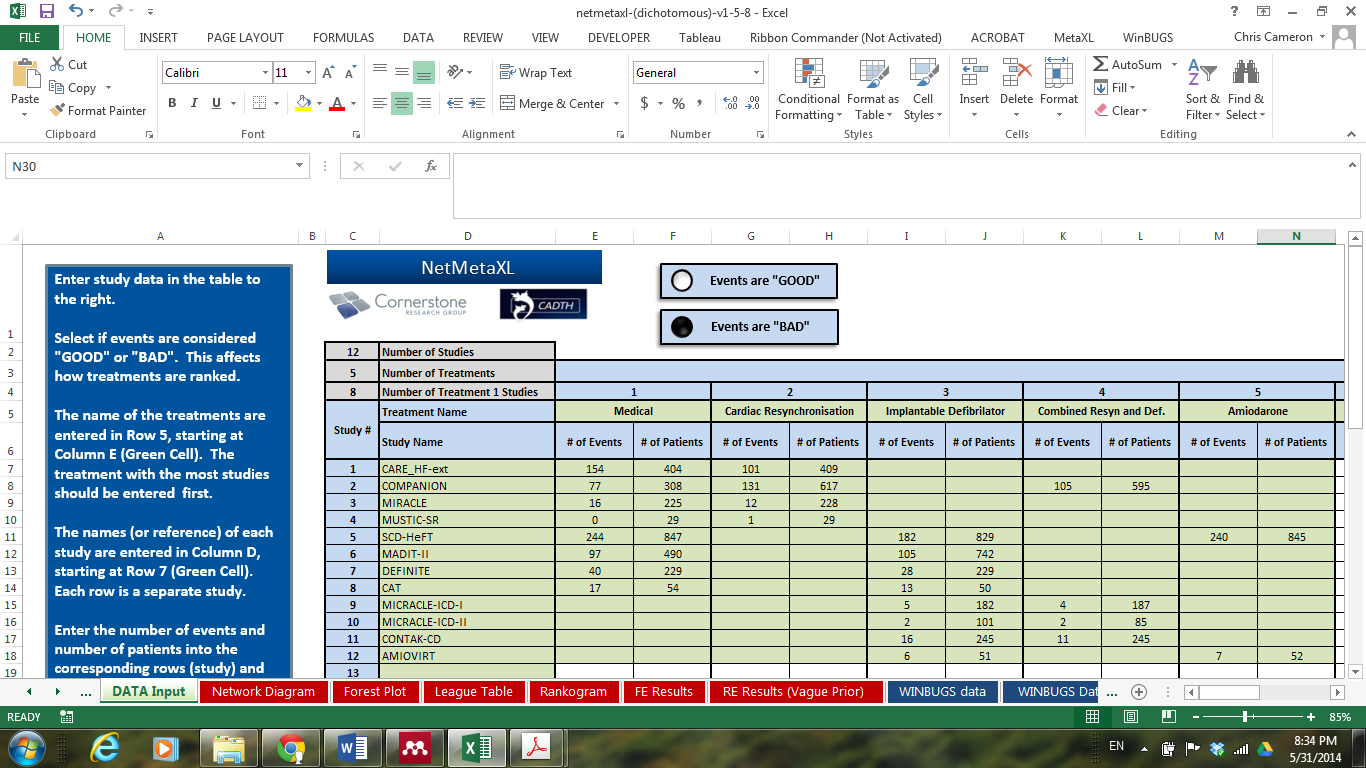


**Figure 2:** Screenshot of evidence network diagram


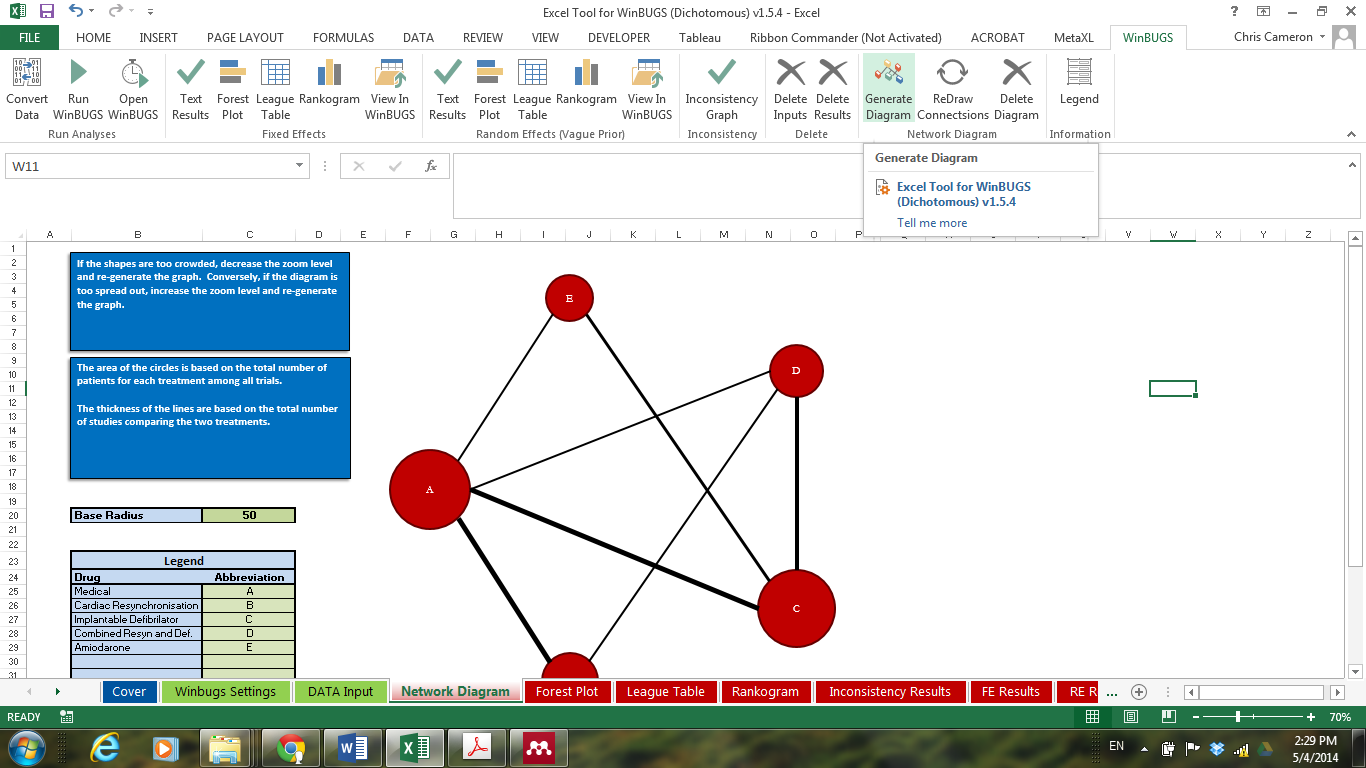


**Figure 3:** Screenshot of analysis dialogue box


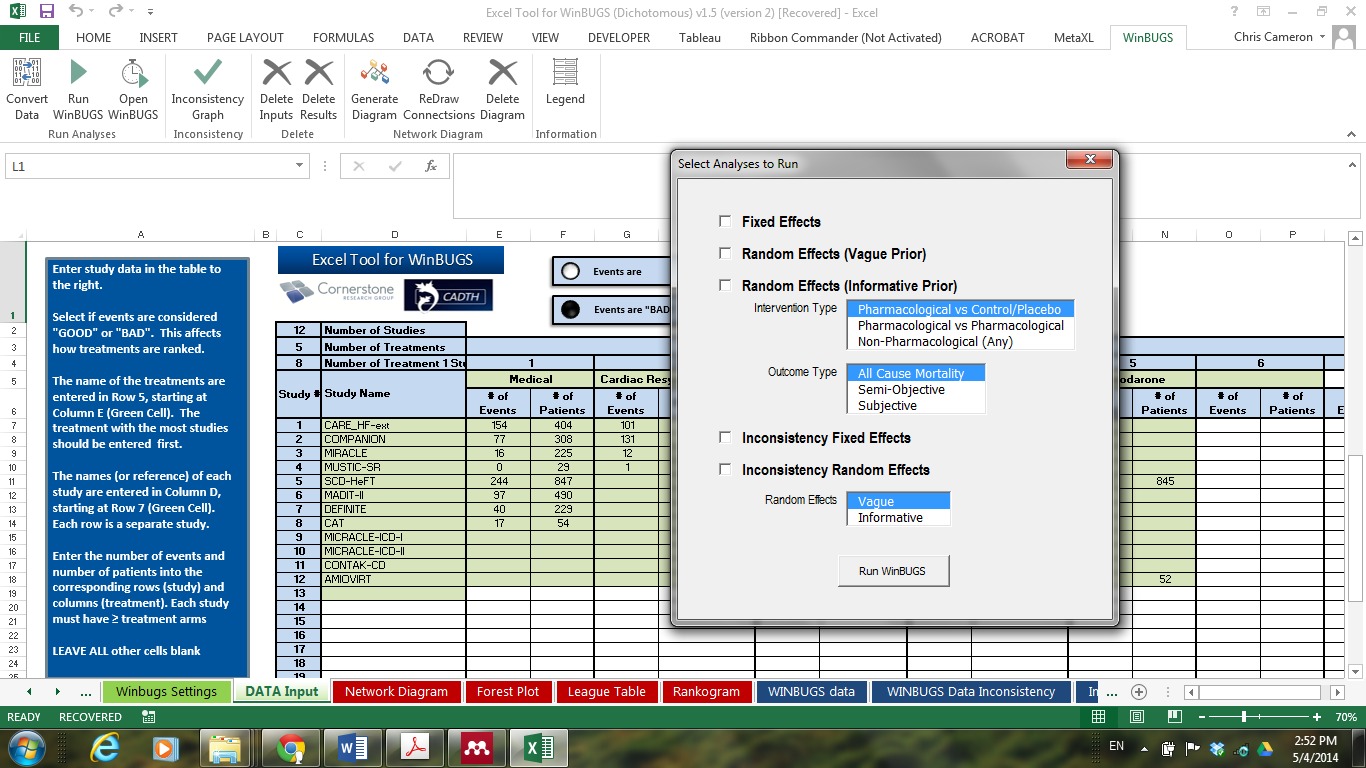


**Figure 4:** Screenshot of forest plots


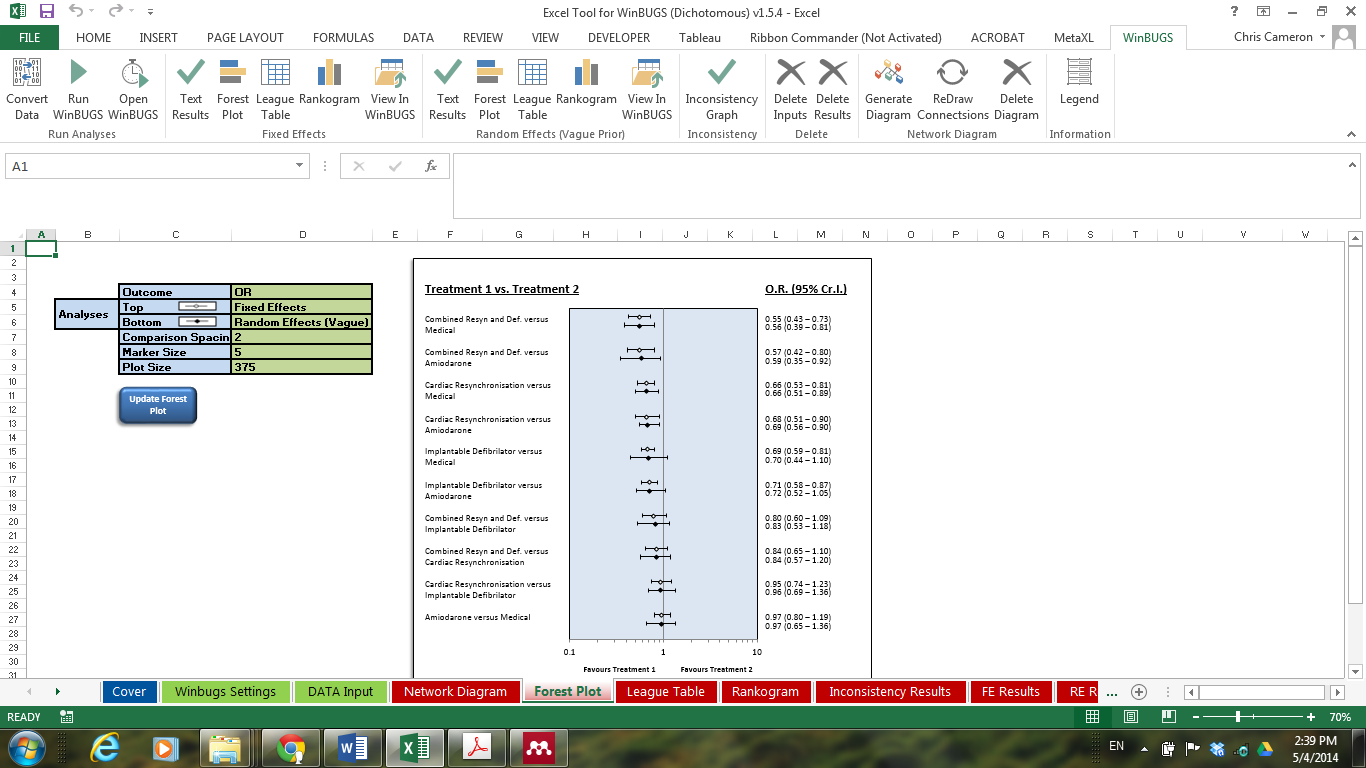


**Figure 5:** Screenshot of league table


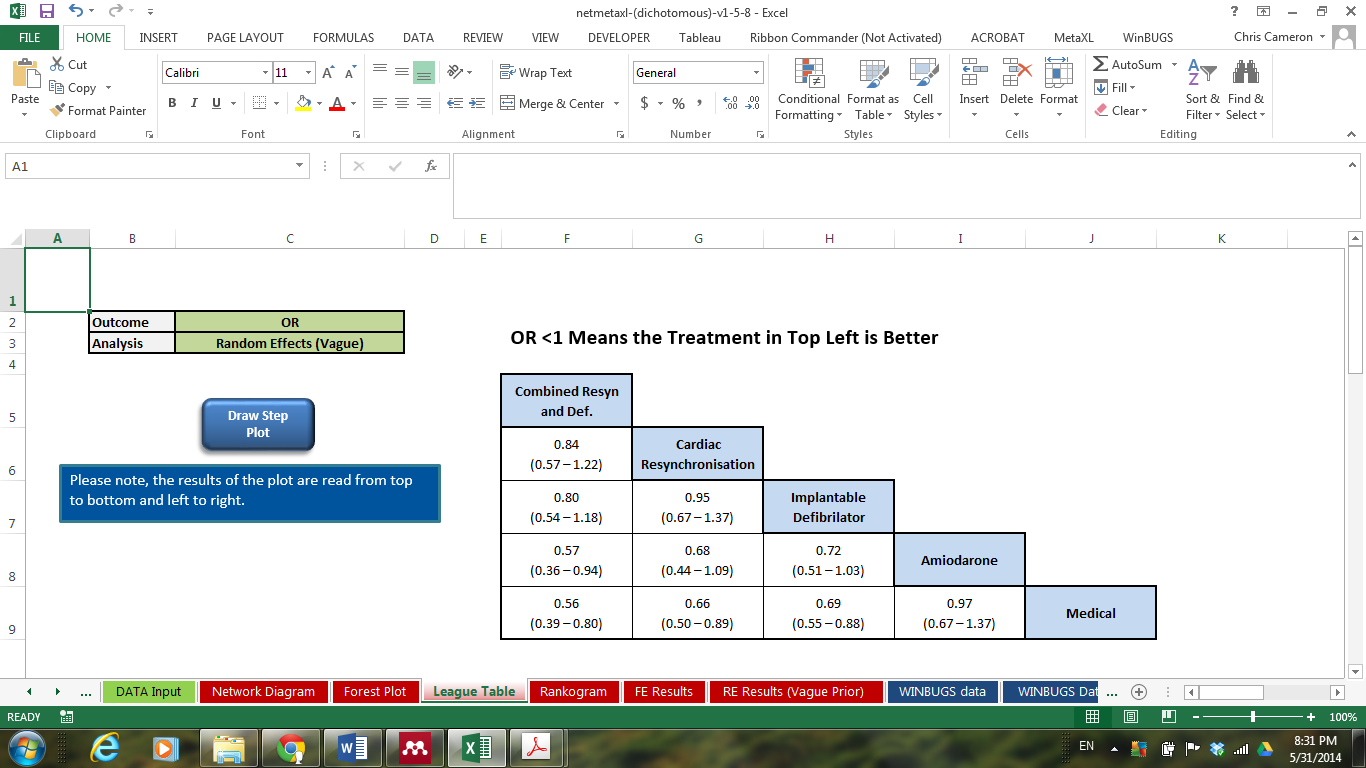


**Figure 6:** Screenshot of inconsistency plots


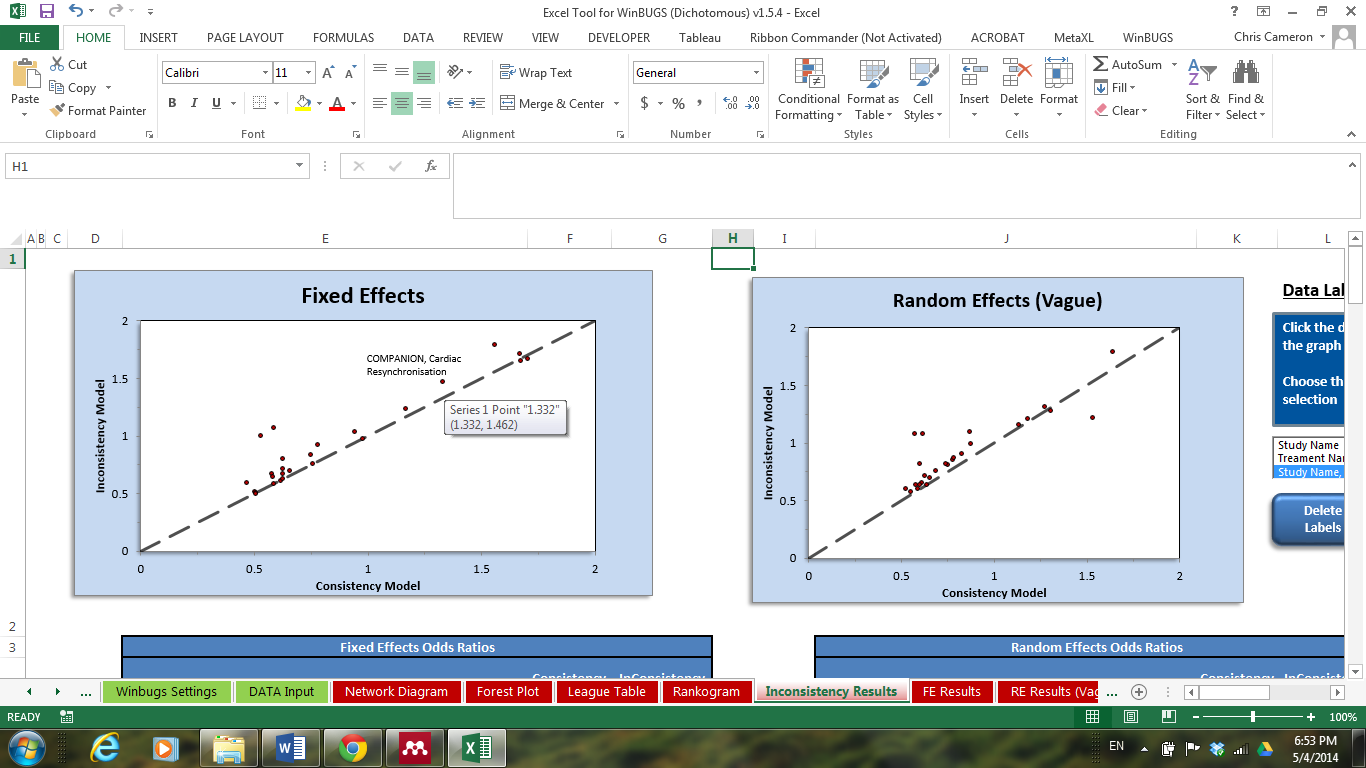

Supplement: Additional file 1 — Screenshots of NetMetaXL. Multiple screenshots for selected steps within NetMetaXL: data input, generating network diagram, using dialogue box, generating forest plots, generating league tables, and generating inconsistency plots. [file 2046-4053-3-110-S1.docx]
