# Supplementary material for: A Microsoft-Excel-based tool for running and critically appraising network meta-analyses—an overview and application of NetMetaXL
Source: Syst Rev. 2014 Sep 29;3:110. doi: 10.1186/2046-4053-3-110 (PMC4195340; doi:10.1186/2046-4053-3-110)
Supplement: Additional file 2 — Output from WinBUGS. Presentation of traditional output from WinBUGS for illustrative example—network meta-analysis evaluating combined resynchronization and implantable defibrillator therapy in left ventricular dysfunction [8]. [file 2046-4053-3-110-S2.docx]

**Additional file 2 – Output from WinBUGS**

**node mean sd MC error 2.5% median 97.5% start sample**

OR[1,2] 0.671 0.1178 0.005622 0.4901 0.657 0.939 1001 3000

OR[1,3] 0.7032 0.09332 0.004547 0.5369 0.6969 0.906 1001 3000

OR[1,4] 0.5781 0.1144 0.005892 0.4042 0.5654 0.8359 1001 3000

OR[1,5] 0.99 0.1997 0.007204 0.6411 0.9816 1.407 1001 3000

OR[2,3] 1.075 0.2182 0.01034 0.6852 1.062 1.535 1001 3000

OR[2,4] 0.8766 0.1848 0.007773 0.5639 0.8617 1.307 1001 3000

OR[2,5] 1.517 0.3998 0.01582 0.8511 1.483 2.406 1001 3000

OR[3,4] 0.8335 0.1905 0.01053 0.5499 0.8074 1.296 1001 3000

OR[3,5] 1.418 0.2709 0.01087 0.9268 1.408 2.001 1001 3000

OR[4,5] 1.769 0.4681 0.02058 0.9583 1.721 2.783 1001 3000

SUCRA[1] 0.1197 0.1336 0.005339 0.0 0.0 0.25 1001 3000

SUCRA[2] 0.6858 0.2067 0.009148 0.25 0.75 1.0 1001 3000

SUCRA[3] 0.6166 0.1818 0.009711 0.25 0.5 1.0 1001 3000

SUCRA[4] 0.9042 0.1748 0.008322 0.5 1.0 1.0 1001 3000

SUCRA[5] 0.1737 0.1956 0.008319 0.0 0.25 0.75 1001 3000

best[1] 0.0 0.0 1.054E-12 0.0 0.0 0.0 1001 3000

best[2] 0.1633 0.3697 0.01359 0.0 0.0 1.0 1001 3000

best[3] 0.1077 0.31 0.01636 0.0 0.0 1.0 1001 3000

best[4] 0.717 0.4505 0.02093 0.0 1.0 1.0 1001 3000

best[5] 0.012 0.1089 0.003131 0.0 0.0 0.0 1001 3000

dev[1,1] 1.169 1.514 0.04422 8.484E-4 0.6207 5.314 1001 3000

dev[1,2] 1.185 1.558 0.04034 0.001455 0.564 5.432 1001 3000

dev[2,1] 1.163 1.472 0.06363 0.001649 0.6219 5.2 1001 3000

dev[2,2] 1.142 1.533 0.04587 0.001273 0.5578 5.391 1001 3000

dev[2,3] 0.9624 1.318 0.03317 9.834E-4 0.4827 4.632 1001 3000

dev[3,1] 0.6635 0.9119 0.01502 5.727E-4 0.3143 3.259 1001 3000

dev[3,2] 0.609 0.8667 0.01866 7.838E-4 0.2785 3.103 1001 3000

dev[4,1] 1.214 1.227 0.01936 0.0338 0.8374 4.831 1001 3000

dev[4,2] 1.807 2.019 0.03611 0.00525 1.153 7.102 1001 3000

dev[5,1] 0.8195 1.162 0.03188 8.741E-4 0.3801 4.221 1001 3000

dev[5,2] 0.9112 1.306 0.04048 0.001094 0.4017 4.512 1001 3000

dev[5,3] 0.9942 1.349 0.02872 0.001225 0.4624 4.75 1001 3000

dev[6,1] 0.8105 1.2 0.02919 7.935E-4 0.361 4.081 1001 3000

dev[6,2] 0.8634 1.249 0.02686 7.99E-4 0.3898 4.518 1001 3000

dev[7,1] 0.7636 1.091 0.02206 0.001072 0.3557 3.735 1001 3000

dev[7,2] 0.6116 0.8673 0.01802 5.944E-4 0.2728 3.156 1001 3000

dev[8,1] 0.6604 1.006 0.01964 7.741E-4 0.266 3.518 1001 3000

dev[8,2] 0.578 0.826 0.01745 6.248E-4 0.2651 3.077 1001 3000

dev[9,1] 0.6275 0.9177 0.01859 8.56E-4 0.2648 3.319 1001 3000

dev[9,2] 0.5329 0.7529 0.0144 7.362E-4 0.2588 2.587 1001 3000

dev[10,1] 0.5969 0.8309 0.01554 7.593E-4 0.2729 2.956 1001 3000

dev[10,2] 0.6272 0.8507 0.0167 6.774E-4 0.2853 2.904 1001 3000

dev[11,1] 0.8408 1.186 0.03132 8.441E-4 0.3896 4.087 1001 3000

dev[11,2] 0.6804 0.9549 0.02122 7.322E-4 0.304 3.418 1001 3000

dev[12,1] 0.5968 0.8239 0.01395 5.492E-4 0.2703 3.054 1001 3000

dev[12,2] 0.6529 0.8965 0.01518 4.335E-4 0.2992 3.247 1001 3000

prob[1,1] 0.0 0.0 1.054E-12 0.0 0.0 0.0 1001 3000

prob[1,2] 0.002333 0.04825 0.001089 0.0 0.0 0.0 1001 3000

prob[1,3] 0.011 0.1043 0.002483 0.0 0.0 0.0 1001 3000

prob[1,4] 0.4497 0.4975 0.02069 0.0 0.0 1.0 1001 3000

prob[1,5] 0.537 0.4986 0.02072 0.0 1.0 1.0 1001 3000

prob[2,1] 0.1633 0.3697 0.01359 0.0 0.0 1.0 1001 3000

prob[2,2] 0.4877 0.4998 0.02309 0.0 0.0 1.0 1001 3000

prob[2,3] 0.29 0.4538 0.02236 0.0 0.0 1.0 1001 3000

prob[2,4] 0.047 0.2116 0.007524 0.0 0.0 1.0 1001 3000

prob[2,5] 0.012 0.1089 0.003247 0.0 0.0 0.0 1001 3000

prob[3,1] 0.1077 0.31 0.01636 0.0 0.0 1.0 1001 3000

prob[3,2] 0.2823 0.4501 0.01975 0.0 0.0 1.0 1001 3000

prob[3,3] 0.5797 0.4936 0.02602 0.0 1.0 1.0 1001 3000

prob[3,4] 0.02933 0.1687 0.004172 0.0 0.0 1.0 1001 3000

prob[3,5] 0.001 0.03161 7.343E-4 0.0 0.0 0.0 1001 3000

prob[4,1] 0.717 0.4505 0.02093 0.0 1.0 1.0 1001 3000

prob[4,2] 0.2047 0.4035 0.0146 0.0 0.0 1.0 1001 3000

prob[4,3] 0.06033 0.2381 0.009341 0.0 0.0 1.0 1001 3000

prob[4,4] 0.014 0.1175 0.00334 0.0 0.0 0.0 1001 3000

prob[4,5] 0.004 0.06312 0.001458 0.0 0.0 0.0 1001 3000

prob[5,1] 0.012 0.1089 0.003131 0.0 0.0 0.0 1001 3000

prob[5,2] 0.023 0.1499 0.004074 0.0 0.0 0.0 1001 3000

prob[5,3] 0.059 0.2356 0.007799 0.0 0.0 1.0 1001 3000

prob[5,4] 0.46 0.4984 0.02046 0.0 0.0 1.0 1001 3000

prob[5,5] 0.446 0.4971 0.02111 0.0 0.0 1.0 1001 3000

resdev[1] 2.355 2.098 0.07483 0.06381 1.796 7.64 1001 3000

resdev[2] 3.267 2.427 0.0917 0.2688 2.698 9.622 1001 3000

resdev[3] 1.273 1.432 0.02867 0.02867 0.7911 5.186 1001 3000

resdev[4] 3.021 1.638 0.03199 1.451 2.453 7.474 1001 3000

resdev[5] 2.725 2.249 0.06841 0.1856 2.153 8.25 1001 3000

resdev[6] 1.674 1.791 0.0448 0.0331 1.102 6.616 1001 3000

resdev[7] 1.375 1.524 0.0337 0.02753 0.8695 5.631 1001 3000

resdev[8] 1.238 1.554 0.03255 0.0239 0.6787 5.666 1001 3000

resdev[9] 1.16 1.465 0.02982 0.02019 0.6358 5.379 1001 3000

resdev[10] 1.224 1.446 0.02818 0.04785 0.7004 5.241 1001 3000

resdev[11] 1.521 1.618 0.04671 0.03939 1.036 6.156 1001 3000

resdev[12] 1.25 1.449 0.02481 0.02922 0.7463 5.436 1001 3000

rk[1] 4.521 0.5344 0.02135 4.0 5.0 5.0 1001 3000

rk[2] 2.257 0.8267 0.03659 1.0 2.0 4.0 1001 3000

rk[3] 2.534 0.7272 0.03885 1.0 3.0 4.0 1001 3000

rk[4] 1.383 0.6993 0.03329 1.0 1.0 3.0 1001 3000

rk[5] 4.305 0.7823 0.03328 2.0 4.0 5.0 1001 3000

sd 0.1592 0.1118 0.008031 0.0141 0.137 0.4441 1001 3000

totresdev 22.08 6.092 0.1564 12.05 21.53 36.21 1001 3000

dic.stats()

DIC

**Dbar = post.mean of -2logL; Dhat = -2LogL at post.mean of stochastic nodes**

**Dbar Dhat pD DIC**

r 144.068 125.882 18.186 162.253

total 144.068 125.882 18.186 162.253

gr(OR)
